# Fusion weighted features and BiLSTM-attention model for argument mining of EFL writing

**DOI:** 10.3389/fpsyg.2023.1049266

**Published:** 2023-01-25

**Authors:** Jincai Yang, Meng Zheng, Yingliang Liu

**Affiliations:** ^1^School of Computer Science, Central China Normal University, Wuhan, China; ^2^School of Foreign Languages, Wuhan University of Technology, Wuhan, China

**Keywords:** EFL writing, Toulmin’s model, artificial intelligence, argument mining, deep learning

## Abstract

Argument mining (AM), an emerging field in natural language processing (NLP), aims to automatically extract arguments and the relationships between them in texts. In this study, we propose a new method for argument mining of argumentative essays. The method generates dynamic word vectors with BERT (Bidirectional Encoder Representations from Transformers), encodes argumentative essays, and obtains word-level and essay-level features with BiLSTM (Bi-directional Long Short-Term Memory) and attention training, respectively. By integrating these two levels of features we obtain the full-text features so that the content in the essay is annotated according to Toulmin’s argument model. The proposed method was tested on a corpus of 180 argumentative essays, and the precision of automatic annotation reached 69%. The experimental results show that our model outperforms existing models in argument mining. The model can provide technical support for the automatic scoring system, particularly on the evaluation of the content of argumentative essays.

## 1. Introduction

The argumentative writing is the most commonly-practiced genre for college students. It is the writing task in all kinds of English proficiency tests, such as the College English Level Examination, English Professional Level Examination, TOEFL, and IELTS Examination. In argumentative essays, the writers state their point of view with evidence or reasons to convince the reader to accept it.

Argument mining (AM), an emerging field in natural language processing (NLP), aims to automatically extract arguments and the relationships between them in a wide variety of text datasets. It typically involves two subtasks: (1) Argument Component Identification (ACI), which involves identifying the location and components of an argument (i.e., main claims, assertions, and premises), and (2) Relationship Identification (RI), which involves identifying the relationship between two-argument components (i.e., Support, Attack, None; Lawrence and Reed, 2020). Understanding the structure of argumentation allows one to determine not only the author’s position on a controversial issue, but also the reasons to support the position. Previous studies on AM have mainly focused on texts by native English speakers (e.g., Moens et al., 2007; Stab and Gurevych, 2014; Zhang et al., 2022). Compared with texts produced by proficient native English speakers, argumentative essays written by second language learners are less coherent and exhibit features of interlanguage. Despite the fact that non-native English speakers outnumber native English speakers in the world, very few studies have attempted to mine arguments in texts produced by the former.

In this study, we propose a new model in this study using BiLSTM (Bi-directional Long Short-Term Memory) and attention training to learn the annotated essays written by EFL students at the sentence level and text level in order to achieve automatic annotation.

## 2. Related work

### 2.1. Automated writing evaluation

In recent years, artificial intelligence (AI) has been increasingly applied in the teaching of English as a Foreign Language (EFL). For example, AI speech assessment systems, based on speech recognition, natural language processing, and speech assessment technologies, help learners practice speaking and assess their performance (Zou et al., 2021). Automated Writing Evaluation (AWE) programs, an application of AI in the area of writing assessment, have now been implemented extensively in EFL writing instruction (Liu and Kunnan, 2016). By comparing a written text to a large database of writing of the same genre, AWE programs can provide individualized diagnostic feedback on the users’ writing (Tang and Wu, 2017). Li et al. (2015) noted that the corrective feedback on grammar and mechanics provided by the AWE system helps EFL college students improve the accuracy of the writing. The system allows students to revise their essays multiple times until they are satisfied, thus encouraging learner engagement and autonomy (Zhai and Ma, 2021). However, most feedback provided by the AWE system covers language-related issues including grammar and mechanics, while little content-related feedback is provided because of the challenges in automatically identifying the complex structural relationships in essays, particularly argumentative essays. Although the persuasiveness of an argument is the main feature for assessing argumentative essays, the quality of the argument has been rarely studied in research on AWE.

### 2.2. Toulmin model

The model of Toulmin (1958, 2003), proposed by the English philosopher Stephen Toulmin, is considered to be a useful framework and guide for writing argumentative essays. The model consists of six elements of argument, namely claim, data, warrant, backing, modal qualifier, and rebuttal (Toulmin, 1958). Its revised version mainly includes the claim, data, counterargument claim, counterargument data, rebuttal claim, and rebuttal data (Toulmin, 2003). Toulmin’s argumentation model has been employed to examine the persuasiveness and defects in argumentative essays and has also been widely applied in studies on argumentative essays (Nussbaum et al., 2005). Although other theories of argumentation have been proposed, such as pragma-dialectics of Van Eemeren and Grootendorst, 1992 and argumentation schemes of Walton (2013), these counterparts of Toulmin’s model are believed to be sophisticated and not practical for EFL learners (Cheng and Chen, 2009).

Toulmin (1958, 2003) clearly distinguishes six functional elements, making the model more applicable in analyzing arguments. Existing research has shown that the Toulmin model of argument has strong reasoning power (Hitchcock, 2005) and can improve poor structure in argumentative writing (Voss, 2006). Particularly, counterargument is a key element in Toulmin’s argument model (Verheij, 2005; Liu and Stapleton, 2014). By pointing out the errors or weaknesses of the opposing side’s argument through evidence, the author can further strengthen the viewpoint and make the argument more comprehensive. To date, the Toulmin argumentation model has been widely applied in assessing the quality of second language argumentative writing (Qin and Karabacak, 2010; Liu and Stapleton, 2014; Stapleton and Wu, 2015; Aziz and Said, 2020; Qin, 2020).

Since the Toulmin model includes the elements required for effective argumentation, it can be used to evaluate the completeness of the argument structure and demonstrate the argumentation process. In a sense, it embodies the descriptive, analytical, and normative functions of an argument model. In this study, based on the model of Toulmin (1958, 2003), we propose a method that automatically analyzes EFL students’ argumentative essays and thus can be applied in AWE to evaluate such essays and provide content feedback.

### 2.3. Argument mining

Argument mining aims to automatically retrieve arguments and related information from texts. It has been extended from identifying argumentative passages to various Natural Language Understanding (NLU) tasks, including automatically extracting arguments and the logical relationships between them from unstructured argument-related texts, and then identifying argument structures. In the context of the increasing demand for information retrieval and extraction, AM has received more and more attention due to its potential application prospects (Lawrence and Reed, 2020). By parsing the argument structure, AM can help authors discover missing or conflicting components in the arguments and write better argumentative essays (Stab and Gurevych, 2014).

As argumentation is one of the key aspects in law cases, AM tools for legal texts are applicable to both academic and non-academic legal research (Zhang et al., 2022). Moens et al. (2007) developed automatic identification of argumentative structures in legal texts through AM techniques. NLP technologies and text-mining tools, have also been applied in identifying and characterizing the most relevant information in a given scientific discipline (Accuosto et al., 2021). Paul et al. (2020) proposed an unsupervised graph-based ranking method that extracts relevant multi-hop knowledge from a background knowledge resource in scientific texts.

Not only can AM be used for analyzing formal written texts, it can be also applied in the web registers (such as comments, forum posts, or blogs). Compared to legal texts and scientific publications, texts on social networks do not always contain arguments and they may not even have proper syntax or spelling, which pose a great challenge for AM. Biran and Rambow (2011) identified justifications for the claims in blog comments with the use of a large set of connectives. Goudas et al. (2014) proposed a two-step method to identify argumentative components and the boundaries of these components in social media texts. First, each sentence was classified as argumentative or non-argumentative and achieved an accuracy of 77.4%. Second, each argumentative sentence was segmented using a conditional random field (CRF), and their best model achieved an accuracy of 42.4%. Habernal and Gurevych (2015) first attempted to utilize unlabeled online data, including comments, forum posts, blogs, and argumentative newswire articles. The features are obtained by word embedding and clustering methods, and the semi-supervised method is used for AM.

Some studies have accomplished one or two subtasks of AM. For example, Florou et al. (2013) classified text fragments as argumentative or non-argumentative with discourse markers and extracted features from the tense and mood of verbs. The experimental results show that the F1_score reached 76.4%. Nevertheless, all the subtasks need to be considered in AM. Persing and Ng (2016) designed the first end-to-end argument mining model that combines a pipeline approach with Integer Linear Programming (ILP) to optimize multiple subtasks of argument mining simultaneously. Their corpus includes a limited dataset of 90 argumentative essays. Based on a dataset of 402 essays, Stab and Gurevych (2017) constructed an end-to-end argumentation structure parser that identifies argument components (major claim, claim and premise) at the token level and globally optimizes component types and argumentative relations. The results of different subtasks were reported, but the overall performance remains unknown.

Taking the overall performance into account, Wang et al. (2020) proposed a multi-scale mining model to mine the argument elements (major claim, claim, and premise) at the discourse level, paragraph level, and word level. They also designed an effective coarse-to-fine argument fusion mechanism to further improve the precision rate. Song et al. (2020) took a step further and identified four argument components (main claim, claim, premise, and other) in two datasets (a Chinese dataset and an English dataset) with structural sentence positional encodings to explicitly represent sentence positions and inter-sentence attentions to capture sentence interactions and enhance sentence representation. To the best of our knowledge, most existing models are rule-based or feature-based (Persing and Ng, 2016; Stab and Gurevych, 2017), which require considerable manual efforts and are not flexible or robust in cross-domain scenarios. To solve this problem, Ye and Teufel (2021) proposed a new end-to-end approach in which AM is formalized as a dependency parsing problem and uses a modified biaffine model. This approach also unifies all AM subtasks under token-level so that a single neural network can be used. However, as with many other NLU models, the dominant language of most AM models is English and few models can process texts in other languages. To address this issue, Toledo-Ronen et al. (2020) mined arguments in non-English argumentative essays by adopting the English dataset based on machine translation.

Despite the progress made so far, the current AM techniques for argumentative essays have some limitations. Firstly, statistical and machine learning methods generally use manually constructed features to mark arguments. These approaches tend to be cumbersome in the process and have difficulty modeling the argument structure. Secondly, deep learning-based AM methods can automatically extract text features, but most of them analyze each subtask of AM independently. They first identify the boundary of the sentence, then divide arguments into three categories of premise, claim and major claim, and finally divide arguments into support and attack, which reduces the precision rate. These methods only tackle one sub-task, which ignores the intrinsic connection between the sub-tasks. Without training sets, deep learning cannot extract effective information automatically. Thirdly, argumentative mining has mainly focused on argumentative essays written by native English speakers, and little research has been conducted with EFL writing.

To address these issues, this study explores the automatic mining of argument elements in EFL argumentative essays with the Toulmin model by fusing text and sentence features in a deep learning model. Since the model of Toulmin (1958, 2003) is relatively comprehensive with six elements, there is no need for further relationship identification when mining arguments. However, the distinctions among the Toulmin elements are sometimes blurry, some individual element signals are omitted in the text, and the elements are often mixed in the same paragraph, all of which pose challenges to the mining of the Toulmin elements in the essays.

## 3. Dataset and pre-processing

The data for this study includes 180 argumentative essays collected from second-year English major students at a comprehensive university. Each essay was manually annotated with the Toulmin elements by two experienced writing instructors who have been teaching English writing for over 10 years. Both are female, aging 45 and 40, respectively. In the experiment, according to the distribution of the arguments, 162 were selected as the training set and 18 as the test set. The details are shown in Table 1.

**Table 1:** Description of the dataset.

Factor	Train	Test	Number
Claim	828	87	915
Data	907	94	1,001
Counterargument claim	70	8	78
Counterargument data	59	6	65
Rebuttal claim	34	4	38
Rebuttal data	28	3	31
Others	668	78	746
Total	2,594	280	2,874

Before processing the data, each essay was coded with the six Toulmin elements in a word document (as shown in Example 1).

Example 1: In order to alleviate the traffic pressure, measures should be taken to by our government. In my opinion, one of the best ways to solve the problem is to limit the purchase of private-owned cars. **[claim]** The limit use of private cars can not only help relieve the traffic jam, but also is a good way to protect the environment because of the deterioration of air quality and the air pollution in Wuhan. **[data]** Once the car purchase is limited, the number in car ownership will not be increase in Wuhan.**[data]**

All essays were then processed in the following steps.

Step 1: All essays were read carefully and an identifier was added at the end of each paragraph, which prepares for the next step.

Step 2: Each essay was divided into sentences, and each sentence was tagged with one of the six elements of the Toulmin model. The tag None is given to non-arguments. Each sentence was also labeled with the position information, which includes the position of the sentence in the text, the position of the sentence in the paragraph to which it belongs, and the position of the paragraph in the text.

Step 3: The end of last sentence in each essay was tagged to mark the end of the essay and to facilitate subsequent differentiation.

Step 4: Each sentence was annotated with part-of-speech tagging with the Natural Language Toolkit (NLTK).

**Figure 1:**
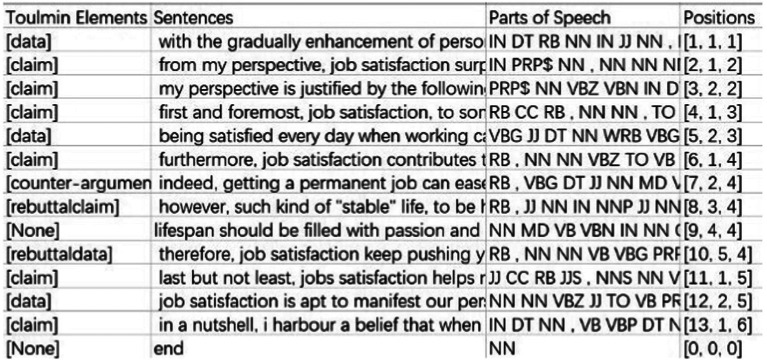
Example of processed text [*Note*: The first two columns indicate the Toulmin elements and corresponding sentences; the third column represents the part-of-speech of each word in the sentence (e.g., IN refers to preposition, RB refers to adverb, NN refers to noun); the fourth column shows the position of each sentence in the text (the first number indicates the position of the sentence in the text, the second number refers to the position of the sentence in the paragraph to which it belongs, and the third number shows the position of the paragraph in the text)].

All processed results were saved in a csv file and an example of processed essay is shown in Figure 1.

## 4. The model and results

### 4.1. Fusion weighted features with BiLSTM attention model

In this study, we tagged the part of speech in the essays with the official version model provided by NLTK. The content in the essay and its part-of-speech tagging were encoded using BERT (Bidirectional Encoder Representations from Transformers). A single-layer BiLSTM (Bi-directional Long Short-Term Memory) was used, the hidden layer dimension was set to 64, the Dropout rate was set to 0.5, and the learning rate was set to 0.001. The optimizer used Adam’s algorithm, and the experiments were iterated 500 times.

BERT was chosen as the word vector model as it is pre-trained using a masked language model and next sentence prediction, which can produce a richer dynamic word vector by making full use of contextual information. LSTM can capture the longer distance dependencies, but cannot encode the information from backward to forward. BiLSTM, composed of forward LSTM and backward LSTM, was therefore chosen for modeling in order to better understand the contextual information.

**Figure 2:**
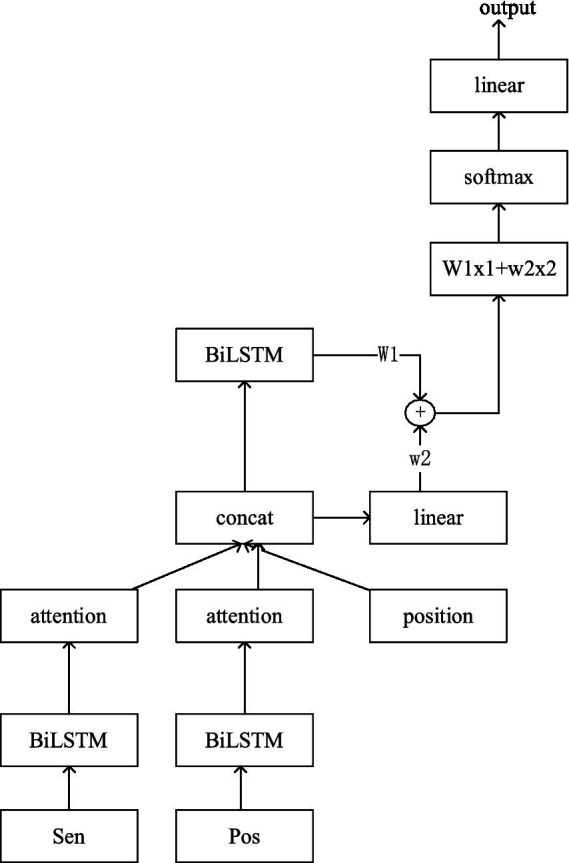
Architecture of the FWFBA model.

The architecture of the fusion Weighted Features with BiLSTM Attention (FWFBA) model proposed in this study is shown in Figure 2. The model consists of three main parts: sentence feature extraction, document feature extraction, and weighted features.

In this model, Sen (sentence) is extracted from the argumentative essay and Pos represents the part-of-speech tagging, both features being obtained by BERT encoding. Position represents the position of each sentence in the text. x1 is the encoding obtained by the linear layer after the feature connects, and x2 is the result obtained after the second BiLSTM encoding.

Our task is to label the sentences (*x_1_,x_2_,…,x_n_*) in the argumentative essay with *Y* = (*y_1_,y_2_,…,y_n_*). *x_i_*(1 ≤ *i* ≤ *n*) is the word in the sentence encoded by BERT (*i* indicates the position of the word in the sentence). *y_i_*(1 ≤ *i* ≤*n*) is the sentence tagged as the argument element in the Toulmin model (*i* indicates the position of the sentence in the essay).

#### 4.1.1. Sentence feature extraction

Each essay consists of $X=\left(X^{1},X^{2},\ldots ,X^{N}\right)$ , in which $\;X^{i}$ represents each sentence and $X^{i}$ =( $x_{1}^{i},x_{2}^{i},\ldots ,x_{n}^{i}$ ). $x_{1}^{i}$ refers to part-of-speech tagged by NLTK. Adding part-of-speech tagging enables the model to better capture semantic information. The sentences and part-of-speech tagging are encoded with BERT. The formula is shown in Eq. (1).


(1) $$X^{i}=BERT\left(X^{i}\right)\;$$

The feature extraction was then performed using BiLSTM and attention mechanism. The BiLSTM model can obtain contextual information based on the semantic and part-of-speech information of the input text, solving the problems of gradient disappearance and gradient explosion. Attention mechanism can focus more on the words or expressions that can indicate a certain argument element. For example, “in my opinion” in Example 2 indicates the claim element.

Example 2: In my opinion, it is a good thing to train animals.**[claim]**

The formulas of the feature extract are shown in Eqs. (2)–(4).


(2) $$h_{i},s_{o}=BiLSTM\left(X^{i}\right)\;$$

(3) $$a_{i}=\mathrm{sotfmax}\left(h_{i}\times \mathrm{concat}\left(h_{i},s_{o}\right)\right)\;$$

(4) $$\mathrm{Attention}\left(X\right)=\mathrm{softmax}\left(h_{i}\times a_{i}\right)$$

Where $a_{i}$ calculates the correlation between $h_{i}$ and $s_{o}$ . $h_{i}$ is the output result after BiLSTM, and $s_{o}$ is the last state obtained by $X^{i}$ after BiLSTM. Sen and Pos are then brought into the above equations, respectively, to obtain *X*_Sen_ and *X*_Pos_.

#### 4.1.2. Document feature extraction

The results obtained from parsing each sentence and part-of-speech tagging in each essay in the sentence feature extraction layer were used as the input of the document feature extraction layer. The experimental results show that the precision improved after using part-of-speech tagging as the feature input.

Following Song et al. (2020), we also used the position information as the feature input. The sentence, part-of-speech tagging and position information was then input to BiLSTM for feature extraction as a whole to better understand the contextual information. As shown in Example 3, some Toulmin elements need to be marked by referring to contextual information.

Example 3: Second, high job satisfaction does not mean low salary or changeable life and a permanent job also does not mean a satisfying life. **[claim]** It’s true that a permanent job is always associated with staple life. **[counterargument claim]**

The formulas of the document feature extract are shown in Eq. (5).


(5) $$\;X_{2}=\mathrm{BiLSTM}\left(\mathrm{concat}\left(X_{\mathrm{Sen}}{\mathrm{,X}}_{Pos}\mathrm{,Position}\right)\right)\;$$

#### 4.1.3. Weighted features

Weighted features are the summation of the embedding output from different layers according to their weights.

Since different layers of the network model have different feature distributions and different effects on the results, we assign different weights W to the two layers for different degrees of scaling so as to better integrate the model. The calculation formula is shown in Eq. (6).


(6) $$F=W\times X_{2}+\left(1-W\right)\times \left(\mathrm{concat}\left(X_{Sen},X_{Pos}\right)\right)$$

### 4.2. Classification results and evaluation

**Figure 3:**
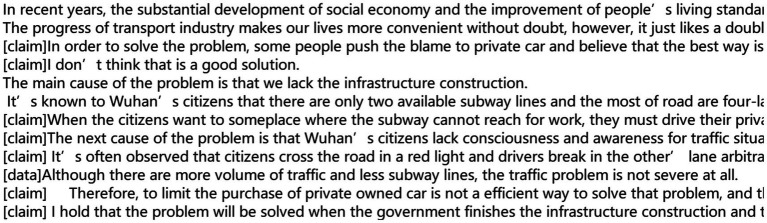
Output of the model.

After feature fusion, the data were first normalized and then passed into a linear layer. A three-dimensional array was obtained after the linear layer, representing the probabilities of each sentence corresponding to different Toulmin elements. After the transformation, the output of the model obtained is shown in Figure 3.

In this study, we use P (Precision) and F1_score as the evaluation indicators. The specific formula of P is shown in Eq. (7), and the formula of F1_score is shown in Eqs. (8) and (9). F1_score considers both precision and recall, allowing them to reach the maximum and a balance. As a measure for classification problems, F1_score is often used as the final measure in machine learning competitions for multi-classification problems.


(7) $$P=\frac{T_{P}}{T_{P}+F_{P}}$$

(8) $$\mathrm{recall}=\frac{T_{P}}{T_{P}+F_{N}}$$

(9) $$F1\_\mathrm{score}=\frac{2*p*\mathrm{recall}}{p+recall}\;$$

*T*_P_ is the number of correctly identified entities, and F_P_ represents the number of incorrectly identified entities. *F*_N_ is the total number of samples which were predicted to be in one of these categories but turned out not to be in that category.

### 4.3. Results

To validate the model proposed in this study, the experimental results were evaluated by using precision and F1_score. The precision rate is 69.6%, and F1_score is 64.9%.

To verify the effectiveness of the FWFBA model, we selected two sets of comparison experiments, one extracting only sentence features for argument mining, and the other extracting document features without feature fusion. The results show that the former achieved the precision of 59.2% and F1_score of 50.3%. The FWFBA model increased the precision by 10.4% and F1_score by 14.6%. Compared to extracting discourse features for argument mining, the FWFBA model can increase the precision by 6.1% and F1_score by 7.3%.

The comparison of the experimental results shows that part-of-speech tagging and position information can help mining the arguments. Compared to that of part-of-speech tagging, tagging including both part of speech and position information can increase the precision by 3.2%, the F1_score by 7.3%. Compared to that of position tagging, tagging including both features can increase the precision by 0.7%, the F1_score by 5.9%. This proves that including both part of speech and position information in the tagging can increase the precision to 69.6% and the F1_score reaches 64.9%.

To verify the practicality of the FWFBA model proposed in this study, we tested the model with the dataset constructed by Stab and Gurevych (2017), the largest and most commonly used dataset in argument mining of argumentative essays. A three-layer BiLSTM is used in this experiment. The experimental results were compared with the DiSA model proposed by Song et al. (2020), which encodes location information for sentences in the text. The results demonstrate the effectiveness of location information. The precision and macro-F1 have both improved, which proved the validity of our proposed model. Compared to DiSA, the precision improved by 7.7%, and macro-F1 improved by 4.4% on FWFBA.

Overall, the experimental results show that the proposed argument mining model using weighted feature fusion inter-sentence information and document information can mine and identify argumentative elements more effectively.

## 5. Discussion and conclusion

In this study, we propose an argument mining model for argumentative essays based on a corpus of 180 argumentative essays written by EFL students. Our model performs feature mining at the sentence level and discourse level, combines part-of-speech and location information to mine different types of argumentative components, and then fuses the features into a deep learning. The features are then fused into the deep learning model for argumentative factor mining. By incorporating different features, the best feature fusion is obtained.

The experimental results show that the proposed framework can effectively identify and classify arguments, with a precision of 69.6% and a value of 64.9% on F1-score. In order to verify the effectiveness of the model, we also conducted experiments with a corpus of 402 essays constructed by Stab and Gurevych (2017), and the results show an improvement of 7.7% in precision and 4.4% in macro-F1 compared with Song et al. (2020). This suggests that the model proposed in this study is effective and can better identify the argument structure.

Since the Toulmin model contains rebuttal elements, Argument Component Identification (ACI) and Relationship Identification (RI) can be viewed as a whole task when performing argument mining. Our model performs feature extraction at the sentence level and the chapter level to mine different levels of features. Part-of-speech tagging is incorporated at the sentence level and positional information is incorporated at the discourse level to mine different types of argumentative elements. Then we fused the features at different levels to obtain the best result for mining argumentative elements in argumentative essays. The experimental results demonstrate the effectiveness of our proposed model.

The proposed mining model contains several consecutive tasks, which may lead to potentially erroneous results of the upstream model and further negatively affect the results of the downstream model. Considering this, we classified the sentences in the essays based on the Toulmin model. ACI and RI were treated as one task, which reduced the impact of the errors on the results.

In constructing the model, we have considered the fact that different elements of the argument correspond to different token-lever. For example, claims lead specific paragraphs as the core statements. They can appear anywhere in a paragraph, either proposed at the beginning, summarized in the end, or given in the middle. They are at the paragraph level (Wang et al., 2020). Similarly, the different elements in Toulmin models correspond to token-lever. We first feature-mined the essays on different token-lever, and then fused the mined features with the features. Inspired by the encoding of sentence position structure of Song et al. (2020), we input sentence position information as features into the model. The experimental results show that position information is indeed helpful for AM of argument essays.

Some limitations of the study should be acknowledged. Due to the limited dataset, the automatic learning model cannot quickly identify the implied features; in addition, the limited number of fused texts and sentence features may affect the precision of recognition. Future research can mine more textual features and combine them into a model based on a larger corpus, and use more artificial intelligence computational models to improve the performance.

In future work, we will explore more features of the Toulmin elements and identify information such as the tangibility of the arguments in the text to score argumentative essays. This line of research can facilitate the AWE to provide feedback on the content of argumentative essays and can help students improve argumentative writing.

## Data availability statement

The original contributions presented in the study are included in the article/Supplementary material, further inquiries can be directed to the corresponding author.

## Author contributions

JY and YL conceived of the initial idea and designed the study. YL collected and analyzed the data, and finalized the manuscript for submission as the corresponding author. MZ designed the model and drafted the manuscript. All authors revised subsequent versions and proofread the manuscript. All authors have read and agreed to the published version of the manuscript.

## Funding

This work was supported by a research grant from China Social Science Research Foundation (Grant No: 19BYY229).

## Conflict of interest

The authors declare that the research was conducted in the absence of any commercial or financial relationships that could be construed as a potential conflict of interest.

## Publisher’s note

All claims expressed in this article are solely those of the authors and do not necessarily represent those of their affiliated organizations, or those of the publisher, the editors and the reviewers. Any product that may be evaluated in this article, or claim that may be made by its manufacturer, is not guaranteed or endorsed by the publisher.
