# Supplementary material for: Fusion weighted features and BiLSTM-attention model for argument mining of EFL writing
Source: Front Psychol. 2023 Jan 25;14:1049266. doi: 10.3389/fpsyg.2023.1049266 (PMC9905622; doi:10.3389/fpsyg.2023.1049266)
Supplement: Supplementary file 1 [file Table_1.DOCX]

Additional Table 1: Definition and examples of the Toulmin model （Qin and Kabarack, 2010, p. 449）

| Toulmin factor | definition and example |
| --- | --- |
| Claim | definition: An assertion in response to a contentious topic or problem.  example: Smoking in public is harmful |
| Data | definition: Evidence to support a claim. It can take various forms, such as facts, statistics, anecdotes, research studies, expert opinions, definitions, analogies, and logical explanations.  example: Research has shown that smoking is one of the causes of air pollution. (research  studies |
| Counterargument  claim | definition: The possible opposing views that challenge the validity of a writer’s claim; these  opposing views can also be supported by data.  example:Several manufacturers propose that smoking in public places should be legal. |
| Counterargument  data | definition: Evidence to support a counterargument claim.  example:So many people are willing to afford more cigarettes, The industry of cigars develops at a high speed, which also contributes to our economy. |
| Rebuttal claim | definition: Statements in which the writer responds to a counter-argument by pointing out the possible weaknesses in the claim, data, or warrant, such as logical fallacies, insufficient support, invalid assumptions, and immortal values.  example:Maybe we can say it is a cultural product or part of life. But it harms our human health. |
| Rebuttal data | definition: Evidence to support a rebuttal claim.  example:So it should be partly forbidden, especially in public places. |


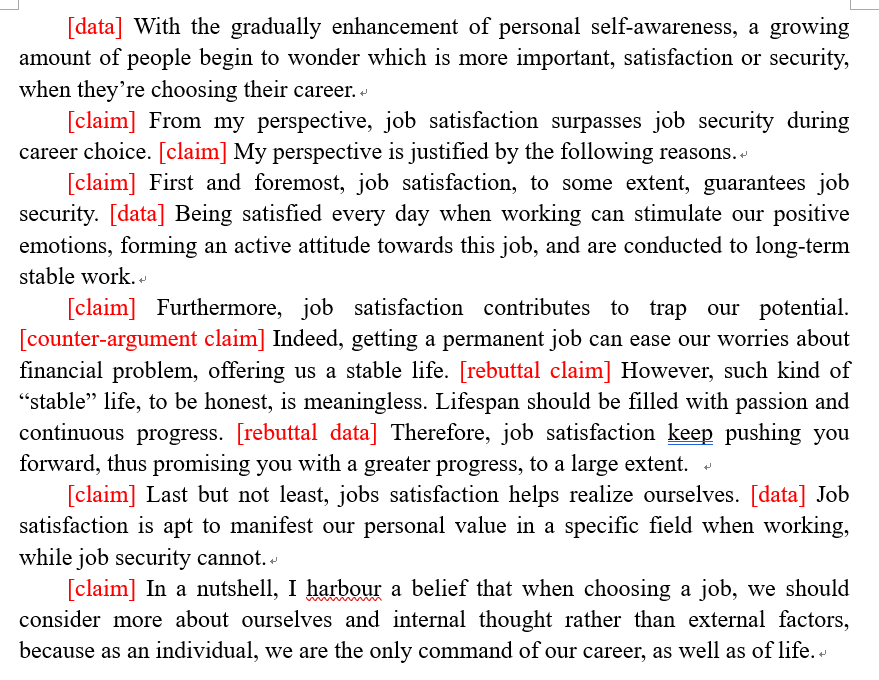


Additional Figure 1 Sample Coded Essay


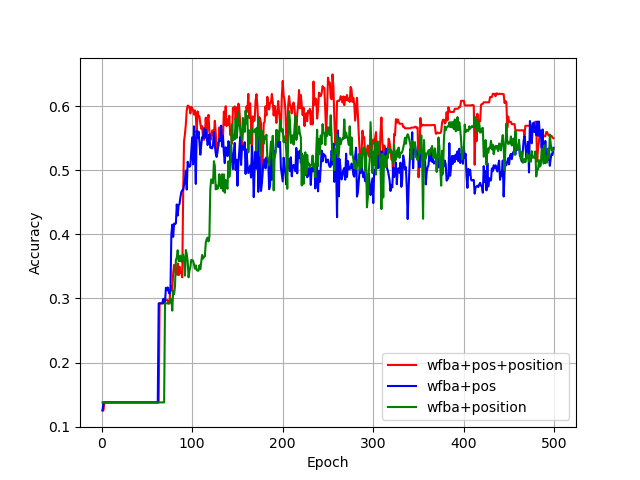


Additional Figure 2 F1_score in Different Conditions
